# Lipid Peroxidation With Implication of Organic Pollution in Autistic Behaviors

**DOI:** 10.7759/cureus.14188

**Published:** 2021-03-30

**Authors:** Kunio Yui, George Imataka, Hitomi Sasaki, Ryoichi Shiroki, Mamiko Koshiba

**Affiliations:** 1 Department of Urology, Fujita Health University Hospital, Toyoake, JPN; 2 Department of Pediatrics, Dokkyo Medical University Hospital, Mibu, JPN; 3 Department of Urology, Fujita Health University, Toyoake, JPN; 4 Department of Anatomy, Graduate School of Science and Technology for Innovation, Yamaguchi University, Ube, 755-8611, JPN

**Keywords:** polyunsaturated fatty acids, lipid peroxidation, autistic behavior, malondialdehyde-modified low-density lipoprotein, docosahexaenoic acid, superoxide dismutase, organic pollution

## Abstract

Background

Lipid metabolism has been associated with the development of autism. The omega-3 and omega-6 polyunsaturated fatty acids (PUFAs) readily undergo lipid peroxidation and conversion to malondialdehyde (MDA). MDA-modified low-density lipoprotein (MDA-LDL) is a marker of lipid peroxidation. However, the association between PUFAs and MDA-LDL in the pathophysiology of autism spectrum disorder (ASD) is unclear.

Materials and methods

We studied the association between PUFAs and MDA-LDL in 16 individuals with ASD (mean age: 11.5 ± 5.7 years) and seven age- and sex-matched healthy controls (mean age: 10.0 ± 4.1 years). The Aberrant Behavior Checklist (ABC) was used to assess behavioral symptoms. We overcame the small sample size by using the adaptive LASSO for enhancing the accuracy of prediction and interpretability. We also estimated the coefficient of variation for an appropriate variable selection and compared additional prior studies to support the findings. Thus, we conducted a careful selection of appropriate candidates to account for confounding variables.

Results

The ASD group had significantly higher plasma MDA levels, eicosapentaenoic acid levels, and a higher ratio of plasma docosahexaenoic acid (DHA)/arachidonic acid (ARA) levels than the control group. Plasma levels of the omega-6 PUFA fraction, dihomo-γ-linolenic acid, and superoxide dismutase levels were significantly lower in the ASD group than in the control group. Total ABC scores were significantly higher in the ASD group than in the control group. Multiple linear regression and adaptive LASSO indicated that plasma DHA levels and plasma DHA/ARA ratios were significantly associated with total ABC scores and plasma levels of MDA-LDL.

Conclusion

Increased plasma levels of DHA and DHA/ARA ratio might be related to organic pollution. These neurobiological bases may induce neuronal deficiency associated with autistic behavioral symptoms in individuals with ASD.

## Introduction

Lipids are the main constituent of cell membranes and play an essential role in maintaining the structural integrity of cells. The oxidative degradation of lipids selectively produces free radicals in the lipid component of cellular membranes [1]. Among the major lipids, polyunsaturated fatty acids (PUFAs) readily undergo lipid peroxidation chain reactions that produce malondialdehyde (MDA) and 4-hydroxy-2-nonenal (4-HNE) [1]. There are a few studies on the association between lipid peroxidation, and environmental and genetic factors. Therefore, such an association may not be scientifically demonstrated. MDA undergoes a chemical reaction with acetaldehyde to form MDA-modified low-density lipoprotein (MDA-LDL). MDA-LDL is a useful biomarker of lipid peroxidation. Previous studies reported significantly higher blood MDA levels in 20 autistic children than age-matched control children [2,3]. Nevertheless, which PUFAs mainly contribute to MDA formation remains a matter of debate.

Accumulating evidence indicated that lipid peroxidation was positively associated with persistent organic pollutant endosulfan and that particulate organics react with macromolecules inducing lipid peroxidation [4]. Organic pollutants induce brain disorders including autism spectrum disorder (ASD) [5]. Thus, in some form, organic pollution may associate with lipid peroxidation in autistic behaviors.

The antioxidant protein superoxide dismutase (SOD) is closely associated with MDA. Serum MDA and SOD levels were increased and decreased, respectively, in 20 children with ASD compared with those in 25 age-matched controls, suggesting that the antioxidant defense mechanism is diminished in individuals with ASD [2]. Serum SOD levels were significantly lower in autistic children younger than controls, whereas MDA was significantly higher in these children than in controls [6]. Thus, the association between lipid peroxidation markers and SOD was still debatable. Therefore, the relationship between MDA and SOD in the pathophysiology of ASD is the subject of ongoing research.

One of the lipid profile components, LDL, plays an important role in brain development. A previous study reported significantly higher LDL levels in 22 adults with Asperger syndrome (mean age: 40.8 ± 10.8 years) than in 22 age-matched controls [7]. Therefore, blood LDL levels might contribute to the pathophysiology of ASD. Recent evidence indicates that MDA-LDL is a good marker of oxidative stress. However, MDA-LDL's role in the pathophysiology of ASD has not been investigated in detail.

Taken together, current evidence supports the following avenues of investigation: (1) which type of omega-3 or omega-6 contributes to MDA formation, (2) the role of MDA-LDL in the pathophysiology of ASD, and (3) the association between lipid peroxidation and antioxidant SOD with respect to autistic behavioral symptoms. Additionally, this study is the first to investigate the association between lipid peroxidation and organic pollution.

This study of a small sample assessed which PUFA contributes to lipid peroxidation products (MDA-LDL) and whether PUFAs and MDA-LDL can predict autistic behaviors. Therefore, to enhance the accuracy of prediction and interpretability, we used the adaptive LASSO [8].

## Materials and methods

Participants

This study included 23 young, physically healthy individuals. Diagnoses were performed based on the Diagnostic and Statistical Manual of Mental Disorders, Fifth Edition (DSM-5) criteria and confirmed using the Autism Diagnostic Interview-Revised. Among the 23 individuals, 16 had an independent clinical diagnosis of ASD and the remaining seven were normal healthy controls. The 16 individuals with ASD had the core symptoms as per the DSM-5 diagnostic criteria for ASD without any abnormal neurological symptoms (nine males and seven females; mean age: 11.5 ± 5.7 years; range: 6 to 21 years), and the remaining seven were diagnosed as normal healthy controls (four males and three females; mean age: 10.0 ± 4.1 years; range: 5 to 21 years). None of the ASD or control individuals had a history of other neurological conditions or comorbid psychiatric disorders. The two groups were matched with respect to feeding habits, age, and full intelligence quotient (IQ) scores (Table 1). Although the 16 individuals with ASD received medical treatment, this study's blood sampling procedure was conducted before the medical treatment. Therefore, blood samples were collected without any medical treatment. During the screening, physical (blood pressure and heart rate) and clinical laboratory examinations (hematology and plasma chemistry, including plasma fatty acids, cholesterol, and triglycerides) were performed on all 23 participants. No participants had any abnormalities in their physical examination and laboratory findings. The IQ was estimated using the Wechsler Intelligence Scale for children and adolescents aged 6 to 16 years (i.e., the Wechsler Intelligence Scale for Children-III) or the respective scale for adults (Wechsler Adult Intelligence Scale) (Table 1).

**Table 1:** Subject characteristics and plasma levels of PUFAs, MDA-LDL, and antioxidant proteins, and the ABC total scores. ASD, autism spectrum disorder; ADI-R, Autism Diagnostic Interview-Revised; DHA, docosahexaenoic acid; ARA, arachidonic acid; DGLA, dihomo-γ-linolenic acid; MDA-LDL, malondialdehyde-modified low-density lipoprotein; Tf, transferrin; SOD, superoxide dismutase; Cp, ceruloplasmin; PUFAs, polyunsaturated fatty acids; ABC, Aberrant Behavior Checklist *These indicated significant difference in the 16 individuals with ASD compared to the seven control subjects.

Variables	ASD (n=16)	Controls (n=7)	U	p-Value
Age (years)	11.5 ± 5.5	10.0 ±4.1	45.00	0.49
Sex (male/female)	12/16	4/16	χ^2^=0.30	0.59
Scores of ADI-R				
Domain A (social)	13.8 ± 5.5	N/A		
Domain 2 (communication)	13.4 ± 6.2	N/A		
Domain 3 (stereotyped)	4.8 ± 5.0	N/A		
Plasma biomarker levels				
DHA	3.49 ±1.00	2.52 ± 0.90	28.50	0.07
DHA/ARA	0.58 ± 0.17	0.37 ± 0.08	11.50	0.01*
DGLA (mg/dL)	1.29 ± 0.33	1.60 ± 0.22	25.50	0.04
MDA-LDL (mg/dL)	91.06 ± 17.80	71.29 ± 17.87	24.50	0.03*
Tf (mg/dL)	276.88 ± 44.92	262.29 ± 25.75	43.00	0.41
SOD (U/mL)	2.52 ± 0.48	5.69 ± 4.64	18.00	0.01*
Cp	27.18 ± 5.78	24.29 ± 7.25	44.50	0.45
Total scores of the ABC	51.63 ± 23.56	0.71 ± 1.25	0.00	p < 0.001

This study was conducted with the ethical approval of Dokkyo Medical University, Tochigi, Japan. Written informed consent was obtained from the participants and/or their parents.

Precautions for mitigating the effects of small sample sizes

Interpretation of the results might be limited due to the small sample in this study. No single statistical method can be applied to all settings, and the importance of selecting the method that corresponds most appropriately to the specific design of the study is paramount. Therefore, we applied some measures to overcome statistical restraint in interpreting the data. First, the modified adaptive LASSO is useful for selecting appropriate covariates for inclusion in propensity score models, accounting for confounding bias and maintaining statistical efficiency. We applied the adaptive LASSO to identify the most effective variables among plasma levels of MDL-LDL, docosahexaenoic acid (DHA), eicosapentaenoic acid (EPA), arachidonic acid (ARA), omega-3/omega-6 ratio, and SOD. Second, we expressed variability as standard deviations (SDs) because they better measure the variability or dispersion of individual data values to the mean than standard error. Data reliability was calculated in terms of coefficients of variation (CV), defined as SD/mean values to determine the relative variation of a random variable for assessing and comparing the performance of analytical techniques and equipment.

Evaluation of behavioral symptoms

Behavioral symptoms were assessed using the Aberrant Behavior Checklist (ABC) that captures a wide variety of behavioral problems and can discriminate between disruptive behavior disorders and the behavioral symptoms of ASD.

Assessment of plasma levels of PUFAs, ceruloplasmin, superoxide dismutase, transferrin, and malondialdehyde-modified low-density lipoprotein

Procedures of blood sampling

Blood samples were collected by venipuncture into ethylenediaminetetraacetic acid tubes after five-hour fasting and immediately placed on ice. Regarding fasting time, a previous study indicated that fasting for eight hours was needed [9]. However, plasma lipid profiles can be determined in non-fasting blood samples [10]. Therefore, the five-hour fasting applied herein should be reasonable. After 20 minutes of supine rest in a quiet room, the participant's blood was collected from 11:00 AM to 12:30 PM to reduce the effects of circadian variation. The samples were frozen at -80°C until plasma levels of PUFA, MDA-LDL, and antioxidant proteins were analyzed at a clinical analytical laboratory (SRL Inc., Tokyo, Japan).

Plasma levels of PUFAs

After transmethylation with hydrochloric acid in methanol, the composition of PUFAs was assayed using gas chromatography (GC2010, Shimadzu Co., Kyoto, Japan). The intra- and inter-assay coefficients of ARA were 110.14 μg/mL (SD: 3.87; CV, 5.28%) and 100.63 μg/mL (SD: 5.51; CV: 5.48%), respectively, and those of DHA were 73.87 μg/mL (SD: 2.30; CV: 3.11%) and 68.07 μg/mL (SD: 2.30; CV: 3.33%), respectively. The plasma levels were expressed as mean ± SD weight (%) of the total PUFAs.

Plasma levels of ceruloplasmin

For estimation of plasma ceruloplasmin levels, a Bering BN Ⅱ Nephelometer (Siemens Healthcare Diagnostics KK, Tokyo, Japan) was used. The assay sensitivity was 3.0 mg/dL.

Plasma levels of superoxide dismutase

Plasma SOD levels were estimated from the rate of decrease in nitrite produced by hydroxylamine and the superoxide anions based on the nitrite method using a Versa max instrument (Molecular Devices Co., Tokyo, Japan). Human plasma was assayed using a SOD Assay Kit (Takara Bio, Tokyo, Japan) according to the cytochrome C method. The plasma SOD levels are expressed as units per milliliter. The assay sensitivity was 0.3 U/mL. The intra-assay and inter-assay coefficients were 2.11 U/mL and 2.10 U/mL, respectively.

Plasma levels of transferrin

A standard turbidimetric assay and an automated biochemical analyzer (JCA-BM8000 series, JEOL Ltd., Tokyo, Japan) were utilized to estimate plasma transferrin levels.

Plasma levels of malondialdehyde-modified low-density lipoprotein

An enzyme-linked immunosorbent assay was used to measure plasma levels of MDA-LDL. The plasma samples were mixed with a stabilizing buffer. The detection limit was 6.3 U/L, and intra- and inter-assay coefficients were <5.6% and <9.4%, respectively.

Management of dietary intake and assessment of nutrient intake

As plasma fatty acid levels may be confounded by prior dietary intake, all subjects received the "Japanese Food Guide." To assess the daily food and nutrient intake, a semi-constructive questionnaire for the Japanese was performed using the junior high school version of the DHQ software (DHQ15; DHQ Support Center, Tokyo, Japan).

Statistical analyses

Significant differences between the ASD and control groups were determined using nonparametric Mann-Whitney U tests. Relationships between plasma PUFA and the two groups of participants, and social responsiveness scale scores were confirmed by multiple regression analysis (Table 2). The most effective variables among plasma levels of MDL-LDL, DHA, EPA, and ARA, omega-3/omega-6 ratios, and SOD as a measure for caution in interpreting small sample data were identified using adaptive LASSO. This offers consistency in variable selection and is essential in identifying important variables in small sample sizes [8]. Data were statistically analyzed using IBM SPSS Statistics for Windows, Version 23.0 (IBM Corp., Armonk, NY, USA).

**Table 2:** Results of linear regression analysis DHA, docosahexaenoic acid; ARA, arachidonic acid; DGLA, dihomo-γ-linolenic acid; MDA-LDL, malondialdehyde-modified low-density lipoprotein; SOD, superoxide dismutase; ABC, Aberrant Behavior Checklist; ASD, autism spectrum disorder *Significant contribution to DHA/ARA. **Significant contribution to MDL-LDL.

Model	Model R^2^	Model p-Value	Coefficients		
			B	Beta coefficients	p-Value
DHA/ARA	0.995	0.001			
DGLA			-0.210 ± 0.029	-0.421	0.002*
MDA-LDL			0.003 ± 0.001	0.326	0.007*
SOD			-0.010 ± 0.006	-0.181	0.173
ABC total score			0.000 ± 0.000	-0.081	0.182
Group (1 = ASD, 2 = control)			0.086 ± 0.028	0.234	0.039*
DHA	0.995	0.000			
DGLA			0.996 ± 0.549	-0.102	0.144
MDA-LDL			-0.016 ± 0.006	-0335	0.047**
SOD			0.008 ± 0.050	0.023	0.895
ABC total score			0.002 ± 0.003	0.074	0.394
Group (1 = ASD, 2 = control)			-0.429 ± 0.277	-0.201	0.197

## Results

Study population

Age did not significantly differ between the two groups of participants. The 16 individuals with ASD showed restricted and stereotypical behavior patterns (n = 10) or irritability and crying (n = 6). Their mean total ABC score was 51.63 ± 23.56 (Table 1). As a total ABC score of 60.14 for males aged 13-27 years reflects moderate-to-severe ASD [11], the behavioral symptoms of the 16 patients in this study were moderate.

Assessment of dietary intake

There were no significant differences in intake of cholesterol, protein, carbohydrate, fat, animal fat, and unsaturated fatty acids. Moreover, intake of omega-6 and omega-3 PUFAs, ARA, and DHA did not significantly differ between the ASD and control groups.

Plasma levels of lipid peroxidation-related biomarkers

Plasma MDA-LDL levels were significantly higher, and plasma SOD levels were significantly lower in the ASD group than in the control group. Plasma levels of the n-3 PUFA EPA and the ratio of plasma DHA/ARA were significantly higher, and the plasma levels of omega-6 PUFA family dihomo-γ-linolenic acid (DGLA) were significantly lower in the ASD group than in the control group (Table 1).

Predictor variables: results of multiple linear regression analysis

Multiple linear regression analysis revealed that the plasma DHA/ARA ratio (R^2^ = 0.996, p = 0.000) and plasma DHA levels (R^2^ = 0.995, p = 0.001) were significantly associated with adjustment in the variables, SOD, and the total ABC scores in the two subject groups (Table 3). These findings revealed that plasma ARA and DHA levels and plasma DHA/ARA ratios may predict these variables in the two groups. The use of plasma DGLA levels as the dependent variable showed a significant contribution of the plasma DHA/ARA levels (unstandardized coefficients, B = -0.210 ± 0.029, β = -0.421, p = 0.02) (Table 2). Therefore, the plasma DHA levels and plasma DHA/ARA ratio fit the models that distinguish the ASD group from the control group.

**Table 3:** Results of adaptive Lasso SE, standard error; ABC, Aberrant Behavior Checklist; DHA, docosahexaenoic acid; ARA, arachidonic acid; MDA-LDL, malondialdehyde-modified low-density lipoprotein

	Estimate	SE	p-Value	95% confidence interval	
				Lower bounds	Upper bounds
ABC total scores					
DHA	136.54	54.80	0.013	29.11	243.90
ARA	-104.84	40.24	0.009	-83.70	-25.98
DHA/ARA	-74.88	46.97	0.111	-66.94	17.18
α-linolenic acid	-75.88	26.21	0.004	-126.85	-24.12
MDA-LDL					
DHA	-105.88	50.74	0.036	-205.32	-0.429
DHA/ARA	98.81	32.24	0.002	35.67	161.95
ARA	95.24	39.100	0.015	18,612	171.88
γ-linolenic acid	-41.87	31.30	0.181	-103.22	19.48

Results of adaptive LASSO

We conducted LASSO to enhance the statistical model's predictive accuracy and interpretability for small samples [8]. DHA (coefficient: 136.50), ARA (coefficient: -104.84), and plasma DHA/ARA ratios (coefficient: -74.88) were selected for ABC total scores (Table 3). Plasma levels of DHA (coefficient: -105.88) and plasma ratios of DHA/ARA (coefficient: 98.81) were selected for plasma MDA-LDL levels (Table 3). Collectively, plasma DHA levels and plasma DHA/ARA ratios were more significantly associated with total ABC scores and plasma MDA-LDL levels.

To summarize, the findings on the two statistics indicated that plasma levels of DHA and plasma DHA/ARA ratios are significantly associated with total ABC scores and plasma MDA-LDL.

Coefficients of variation

The mean CVs for plasma PUFA were 0.168 (16.87%) and 0.3325 (33.25%) in the ASD and control groups, respectively.

## Discussion

Due to the small sample size, two measures were taken in the present study. The adaptive LASSO determined the most effective variables of plasma levels of variables (e.g., MDL-LDL, DHA, EPA, ARA, SOD, and plasma ratio of omega-3/omega-6 ratio) and selected useful targets for reducing target variables in small sample studies. In this study, the mean CVs for plasma PUFA were 0.168 (16.87%) and 0.3325 (33.25%) in the ASD and control groups, respectively. The mean CV of 11 children and 18 adolescents with attention-deficit/hyperactivity disorder and 11 healthy adults was 7.8% to 17.7% [12]. Additionally, CV in 115 estimates of health surveys conducted in São Paulo, Brazil, was 20% to 30% [13]. Thus, our variable selection was appropriate in the present study. These results collectively suggested that the approaches used herein allow for accurate interpretation of the data.

Plasma MDA-LDL levels and the plasma DHA/ARA ratio were significantly higher, whereas those of SOD and the n-6 PUFA fraction, DGLA, were significantly lower in the ASD group than in the control group (Table 1). Notably, multiple linear regression analysis and the adaptive LASSO identified plasma DHA levels and the plasma ratio of DHA/ARA as appropriate variables that distinguished the ASD group from the control group.

PUFAs are susceptible to peroxidation by oxygen free radicals, and, upon peroxidation, they produce 4-HNE from ARA and 4-hydroxyhexenal (4-HHE) from DHA [14]. The perceived cytotoxic and hormetic effects of 4-HNE from ARA and 4-HHE from DHA in the central nervous system have been implicated in neurological disorders [14]. Moreover, increased 4-HHE levels may contribute to the development of ASD [14]. DHA is profoundly susceptible to oxidative stress in vitro and plays an important role in signaling and lipid mediator production. DHA promotes synaptic connectivity and cortical processing and is a prospective therapeutic agent for neurodegenerative diseases as it can cross the blood-brain barrier.

Furthermore, DHA also promotes synaptogenesis and synaptic expression of synapsin, which induces positive neuronal plasticity effects [15]. Collectively, DHA plays an important role in lipid peroxidation, signal transduction, and neurological disease prevention. In rat brain tissue, DHA changes the oxidant/antioxidant balance by increasing SOD and reducing MDA-LDL, suggesting reciprocal balance between plasma SOD and MDA-LDL levels. Thus, plasma DHA levels might be related to the reciprocal balance (i.e., increased plasma MDA-LDL and decreased plasma SOD levels).

With respect to the role of ARA in brain function, ARA synthesizes cyclooxygenases (COX), in which COX-2 plays regulation of prostaglandin signaling in modulation of hippocampal synaptic transmission and plasticity [16]. ARA's release induces lipid peroxidation through cytosolic phospholipase A2α (cPLA2α), rendering ARA an important signaling lipid in activated immune cells [17]. Importantly, omega-6 fatty acid peroxidation products, such as 4-HNE, have a protective function as signaling molecules that stimulate cell survival [1]. ARA is metabolized by two pathways, leading to prostaglandin formation, of which prostaglandin E2 (PGE2) is an important mediator of synaptic plasticity. PGE2 activates the canonical Wnt signaling pathway, which is crucial to brain development and organization and contributes to the development of ASD [18]. Therefore, ARA plays an important role in lipid peroxidation.

Concerning the difference in lipid peroxidation between ARA and DHA, the release of ARA from phospholipids is mainly catalyzed by cytosolic PLA2 (cPLA2). The activation of cPLA2 and ARA release has been implicated in various neurological disorders and brain injury [14]. The release of DHA through calcium-independent phospholipase A2β regulates important physiological processes, including inflammation, calcium homeostasis, and apoptosis. A metabolic link between DHA and DHA-derived 4-HHE pathway may provide a therapeutic strategy against oxidative damage due to cerebral ischemia and other brain injuries [14]. To summarize, ARA may contribute to neurological diseases, and DHA has a therapeutic role in lipid peroxidation.

The DHA/ARA balance is important to infants' cognitive and behavioral development [19]. We previously showed that a higher plasma omega-3/omega-6 ratio reflecting lower plasma ARA levels reduces plasma levels of the signaling protein ceruloplasmin and might contribute to the pathophysiology of ASD [20]. A previous study indicated that a beneficial DHA/ARA ratio was at least 1.5/1.0 in behavioral development in infancy [19]. Furthermore, the DHA/ARA ratio of 1.4 may useful in neuronal development [21]. In the present study, the average DHA/ARA ratio in the ASD group was 2:5.3. This imbalance in the present study may contribute to the behavioral symptoms and plasma MDA-LDL levels.

Previous studies reported that a fish oil diet with a DHA-rich Schizochytrium species algal meal contains persistent organic pollution levels and that oily fish, a source of omega-3 PUFAs, may contain persistent organic pollutants [22]. As organic pollutant is related to neurodevelopmental disorders including ASD [5], it could be argued that the present findings are the first to reveal the relationship between lipid peroxidation and increased plasma levels of DHA as well as DHA/ARA ratio as organic pollutant [23].

The plasma level of the n-6 PUFA family, DGLA, was significantly lower in the ASD group than in the control group. DGLA is further converted to prostaglandin E1 (PGE1) by inflammatory cells, and the compound possesses anti-inflammatory and anti-proliferative properties [24]. Therefore, decreased plasma DGLA levels may be related to decrease in anti-inflammatory properties in lipid oxidation [25].

MDA may play a role as a signaling molecule and regulate gene expression [1]. Moreover, MDA is an endogenous material of enzymatic and oxygen radical-produced lipid peroxidation. Importantly, DHA elicits MDA production [26]. Thus, as the end-product of ARA and major PUFA decompositions, plasma MDA-LDL might have little effect on behavioral symptoms of ASD.

Plasma SOD levels were significantly reduced in the ASD group than in the control group. Reduced SOD levels in autistic children might indicate the involvement of mitochondrial SOD in ASD pathogenesis [27]. Loss of SOD2 activity may result in various pathological phenotypes in the central nervous system. Taken together, lower plasma SOD levels may contribute to a neuronal deficit in relation to behavioral symptoms in the autism group. However, further detailed studies are needed.

Lastly, as organic pollutants induce brain disorders, including ASD [5], the present findings are the first to reveal that lipid peroxidation in relation to increased plasma levels of DHA, as well as DHA/ARA ratio might be related to organic pollution [22,23].

This study had some limitations. The main lipid peroxidation product, 4-HNE, is considered a second messenger of reactive oxygen species, which is an important factor in stress- and age-associated diseases [28], but it also plays a cytotoxic role in inhibiting gene expression and promoting cell death [1]. However, MDA is a widely used biomarker for lipid peroxidation of n-3 and n-6 PUFAs [1]. Therefore, we used plasma levels of MDA-LDL but not 4-HNE as a biomarker of lipid peroxidation. This study's small sample size may limit the generalization of the present findings to the whole ASD population. Importantly, we applied two measures to mitigate the difficulty in drawing significant conclusions from a small sample in this study. The present findings also suggested a significant correlation between increased plasma levels of MDA-LDL and decreased plasma levels of SOD. A previous review article indicated similar results [2]. The present findings regarding the importance of the plasma DHA/ARA ratio are consistent with those of previous studies [19,21]. Previous studies have indicated that DHA [1] and DHA/ARA ratios [14] are important for lipid peroxidation [14], supporting the present findings. The ratio of control subjects to cases was small (2.3:1). For reference, a previous statistical study reported that a ratio of up to 3:1 to 4:1 induced significant results compared to 1:1 or 2:1 in the case-control genomic study [29], whereas this ratio of experimental group to control group was 2:1 in a previous randomization clinical trial of a new drug [30]. Thus, the ratio in our study is reasonable. Further studies of a larger sample are needed.

## Conclusions

Multiple linear regression and adaptive LASSO revealed that increases in plasma DHA and plasma DHA/ARA ratios were significantly associated with total ABC scores and plasma levels of the lipid peroxidation product MDA-LDL, which counteracted the decrease in plasma SOD levels in the ASD group. Plasma levels of omega-6 PUFA fraction DGLA were significantly decreased in the ASD group compared with the control group. Reduced plasma DGLA levels may be related to reduced anti-inflammatory properties, inducing tendency to increase in inflammatory state related to behavioral symptoms of ASD. The ratio of plasma DHA/ARA levels in this study was not beneficial in brain function. These neurobiological mechanisms might lead to neuronal deficits associated with behavioral symptoms in individuals with ASD. These findings might shed light on the association between lipid peroxidation related to increased plasma DHA levels and organic pollution in autistic behaviors.
